# Supplementary material for: Plasmon enhanced optical tweezers with gold-coated black silicon
Source: Sci Rep. 2016 May 19;6:26275. doi: 10.1038/srep26275 (PMC4872531; doi:10.1038/srep26275)
Supplement: Supplementary Information [file srep26275-s1.pdf]

Supplementary Information

Plasmon enhanced optical tweezers with gold-coated black silicon

Domna G. Kotsifaki, Maria Kandyla,[1] and Pavlos G. Lagoudakis

Supplementary movie

The movie shows bubble formation on an Ag-coated nanostructured silicon substrate.

Effect of polarizability

In order to investigate the effect of the trapped particle polarizability on the wavelength dependence of the trapping efficiency in a conventional optical trap, we derive an analytical expression of the latter using the Rayleigh approximation[S1]:

$$Q = 4\pi^3 NA^3 e^{-1/2}\left(\frac{r}{\lambda}\right)^3\left(\frac{\varepsilon_p - \varepsilon_m}{\varepsilon_p + 2\varepsilon_m}\right) \quad \text{(Eq. S1)}$$

where λ is the wavelength in the surrounding medium ($\lambda = \lambda_o/n_m$, λ_o the wavelength in vacuum and n_m the refractive index of the surrounding medium), NA the numerical aperture of the objective lens, r the particle radius, and ε_p, ε_m the dielectric permittivity of the particle and the surrounding medium, respectively. Eq. S1 has been derived by employing the definition $Q = Fc/n_mP$, where c is the speed of light and P the power of the trapping laser beam. The trapping force, F, is calculated according to Eq. 17 in Ref. S1, for $(x, y, z) = (w_0/2, 0, 0)$ for which the gradient force takes its maximum value. The beam radius at the beam-waist position, w_0, is calculated as $w_0 = \lambda/(\pi\ \mathrm{NA})$.

Figure S1 shows the theoretical trapping efficiency for 400-nm diameter polystyrene beads in deionized water, as a function of the incident trapping laser wavelength. NA = 1.4 has been used in the calculations, which corresponds to the experimental numerical aperture. The trapping efficiency decreases monotonically with the wavelength of the trapping laser beam and does not present a resonant behavior, as the one shown in Fig. 3 of the main paper.

[1] Corresponding author: kandyla@eie.gr

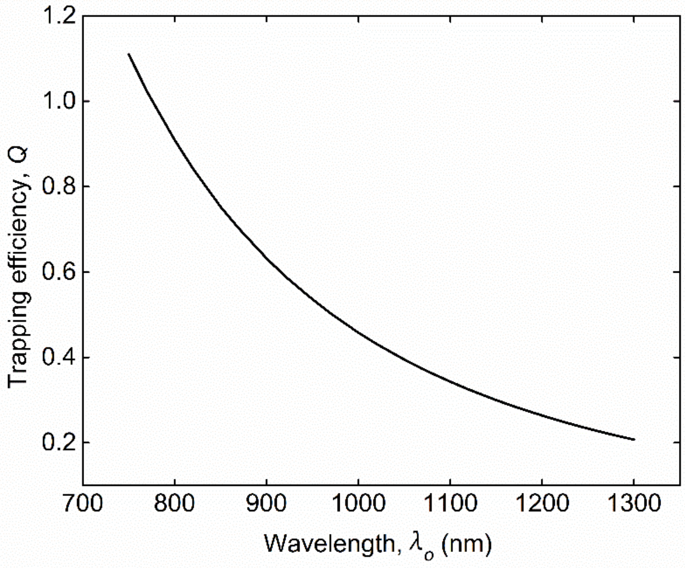

Figure S1: Theoretical trapping efficiency, Q, as a function of the trapping laser wavelength, λ_o.

Exponential decay of trapping efficiency

Figure S2 shows a semilog plot of the trapping efficiency as a function of the relative distance between the trapping laser beam focus and the coated nanostructured silicon substrate, for several trapping wavelengths, below and above the resonance wavelength of 975 ± 30 nm. The data for wavelengths 750 nm, 850 nm, 950 nm, and 1000 nm are offset for clarity purposes. We fit the trapping efficiency data for each trapping laser wavelength with an exponential function, according to the equation $Q = Q_o + Ae^{-bz}$, where Q_o is the experimental trapping efficiency value at a distance z = 10 μm above the coated nanostructured silicon substrate for each trapping laser wavelength and A, b are fitting parameters. The fitting results are shown in Fig. S2 as solid lines, which indicate that as the trapping wavelength approaches the plasmon resonance of the substrate, the exponential decay of the trapping efficiency with the distance from the substrate becomes steeper.

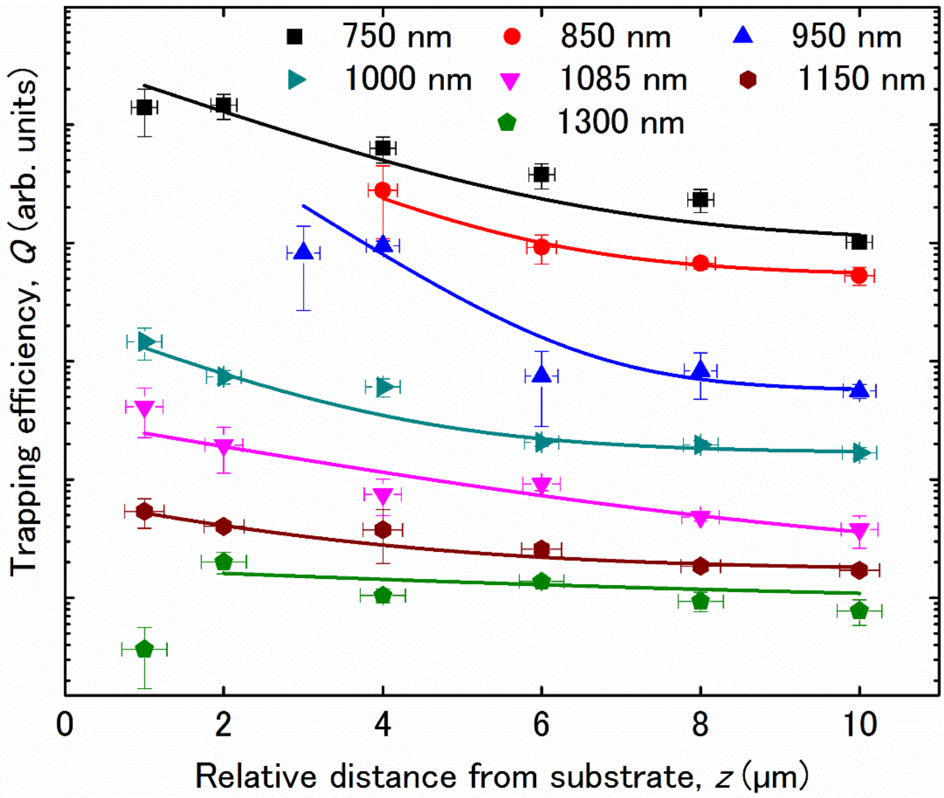

Figure S2: Semilog plot of the trapping efficiency, *Q*, as a function of the relative distance, *z*, between the trapping laser beam focus and the Cu/Au-coated nanostructured silicon substrate, for various trapping laser wavelengths, obtained with the femtosecond laser setup. Relative distance is the measured distance with respect to the zero position. Solid lines: exponential fits to the data. The data for wavelengths 750 nm, 850 nm, 950 nm, and 1000 nm are offset.

References

S1. Y. Harada, T. Asakura, *Opt. Commun.* **1996**, *124*, 529.
